# Factors That Influence Vaccination Decision-Making by Parents Who Visit an Anthroposophical Child Welfare Center: A Focus Group Study

**DOI:** 10.1155/2012/175694

**Published:** 2012-11-20

**Authors:** Irene A. Harmsen, Robert A. C. Ruiter, Theo G. W. Paulussen, Liesbeth Mollema, Gerjo Kok, Hester E. de Melker

**Affiliations:** ^1^Center for Infectious Disease Control, National Institute for Public Health and the Environment (RIVM), P.O. Box 1, 3720 BA Bilthoven, The Netherlands; ^2^Department of Work and Social Psychology, Maastricht University, P.O. Box 616, 6200 MD Maastricht, The Netherlands; ^3^Healthy Living, TNO (Netherlands Organization for Applied Scientific Research), P.O. Box 2215, 2333 AL Leiden, The Netherlands

## Abstract

In recent years, parents have become more disparaging towards childhood vaccination. One group that is critical about the National Immunization Program (NIP) and participates less comprises parents with an anthroposophical worldview. Despite the fact that various studies have identified anthroposophists as critical parents with lower vaccination coverage, no research has been done to explore the beliefs underlying their childhood vaccination decision-making. We conducted a qualitative study using three focus groups (*n* = 16) of parents who visit an anthroposophical child welfare center. Our findings show that participants did not refuse all vaccinations within the Dutch NIP, but mostly refused the Mumps, Measles, and Rubella (MMR) vaccination. Vaccination decisions are influenced by participants' lifestyle, perception of health, beliefs about childhood diseases, perceptions about the risks of diseases, perceptions about vaccine effectiveness and vaccine components, and trust in institutions. Parents indicated that they felt a need for more information. Sufficient references should be provided to sources containing more information about childhood vaccination, especially about the effectiveness of vaccines and vaccine components and the risks, such as possible side effects and benefits of vaccination. This may satisfy parents' information needs and enable them to make a sufficiently informed choice whether or not to vaccinate their child.

## 1. Introduction

In recent years, parents have become more disparaging towards childhood vaccination [1]. Different studies show reasons why parents are critical and sometimes refuse vaccination for their children. These include anxiety about side effects, the perception that vaccine-preventable diseases are not serious, and a lack of trust in herd immunity [2]. This suggests that parents who refuse vaccination are concerned about long-term health problems as a result of vaccination [3] and have doubts about the safety of vaccines [4]. However, these factors vary between different groups of parents who may refuse vaccination. One of the groups who is critical about the National Immunization Program (NIP) and participates less comprises parents with an anthroposophical worldview. 

Anthroposophy is a spiritual movement that was founded at the beginning of the 20th century by Rudolf Steiner, an Austrian philosopher. In anthroposophic care, health is viewed as a matter of body, soul, and spirit, and a balance between these three. There are anthroposophical medical practices in 80 countries around the world [5] and anthroposophy has been applied in various social domains such as education (Steiner schools), art, architecture, and agriculture (biodynamic farming). Various countries have reported lower participation of anthroposophical parents in the NIP including Germany, Sweden, Switzerland, Austria, UK, and The Netherlands [6–11]. Due to the lower vaccination coverage and especially the MMR (Mumps Measles Rubella) vaccination, outbreaks of infectious diseases such as measles still occur among these populations [6, 9–11].

In The Netherlands, the anthroposophical community comprises some 4,300 members [12]. However, the expected number of people with an anthroposophical worldview is higher. Dutch parents can choose whether their children (aged 0–4 years) receive their vaccines and health checkups in a standard child welfare center (CWC) or an anthroposophical CWC. Anthroposophical child vaccine providers (CVPs) are more willing to adapt the NIP if asked to by the parents, compared to CVPs at a standard CWC [13]. The Dutch NIP is managed by the National Institute of Public Health and the Environment (RIVM), is voluntary and free of charge, and has an overall vaccination coverage of 95% [14].

Despite the fact that anthroposophists are identified as critical parents with lower vaccination coverage, no research has been done in The Netherlands and elsewhere to explore their beliefs about childhood vaccination. This qualitative study was conducted, by means of focus group discussions, to gain more insight into parents' experience at an anthroposophical CWC, the factors that influence their vaccination decision-making and their need for information.

## 2. Methods

### 2.1. Participants and Procedure

In total, three focus groups (*n* = 16) were conducted with parents who visit an anthroposophical CWC. Doctors and nurses from three different anthroposophical CWCs in The Netherlands invited parents to participate. Parents received an information letter regarding the study objectives and procedures and could inform the researchers whether they wished to participate by sending an email to an email address. Parents who did so then received more details about the date and location of the focus group discussions.

### 2.2. Study Setting

The focus groups were held in the evening at the anthroposophical CWC that the parents visited and lasted about 2 hours. Every focus group had the same moderator (IH) and different assistants. Informed consent was obtained and participants received a gift voucher of €30 as an incentive. The focus groups were held between May 2011 and June 2012 and were approved by the Psychology Ethics Committee of Maastricht University. The focus groups were based on a semistructured protocol with open-ended questions. The topic list was pilot tested with colleagues and then revised. This revised and final version was used for all the three focus groups.

The focus groups started with an introduction about the objectives of the study and the role of the participants during the focus group. After that, the participants introduced themselves with their names and family composition and reasons why they visited an anthroposophical CWC. Then parents were asked to write down what they perceived as positive and negative aspects of the Dutch NIP. Next, more in-depth questions were asked about which factors influenced their decision whether or not to vaccinate their child, the influence of their social environment in their vaccination decisions, and their need for information. At the end, the focus group was evaluated together with the participants.

### 2.3. Analysis

The three focus groups were recorded with a digital voice recorder and transcribed verbatim. The data were processed with the software program Nvivo 9 (QSR International) and then analyzed based on thematic analysis [15] to explore different factors influencing the parents' decision whether or not to vaccinate their child. Different themes were explored and an inductive process was used to derive subthemes from the six main themes. The data were analyzed and coded by the moderator (IH) and an independent researcher analyzed and recoded one focus group. Afterwards initial coding was compared, reviewed, discussed, and refined until consensus was achieved, leading to an improved coding scheme and criteria.

## 3. Results

### 3.1. Participants

The group of 16 participants consisted of two parents with one child, four parents with two children, another four with three children, one with four children, and one parent with five children. Two couples participated in the focus groups. One of these couples had one child and the other had two. Fourteen of the 16 participants were females. All parents indicated that they had postponed vaccination for at least one child. One parent had refused all vaccinations for her children, while the other parents had partially vaccinated their child. Of the parents who partially vaccinated their children, all refused the MMR, pneumococcal, and meningococcal C vaccinations. The DT-IPV (Diphtheria, Tetanus, and Polio) vaccine was mostly accepted (*n* = 6), next the DTaP-IPV (Diphtheria, Tetanus, Pertussis, and Polio) vaccine (*n* = 3) and then the DTaP-IPV-Hib (Diphtheria, Tetanus, Pertussis, Polio, and *Haemophilus influenzae* type b) vaccine (*n* = 2). The other parents (*n* = 3) had not yet decided whether or not to have their child vaccinated.

The six themes derived from the focus group discussion were divided into sub-themes and are described below with relevant quotes from the participants.

### 3.2. Positive and Negative Aspects of the Dutch NIP

The parents agreed that one positive aspect of the Dutch NIP is that the vaccines are free of charge and available for everyone: “*I think it's good that vaccines are available to everyone, regardless of your background.” *Some participants (*n* = 4) mentioned that some diseases are less prevalent thanks to vaccination. “*The first thing that came to mind was that certain dangerous diseases are less common.”*

One perceived negative aspect of the Dutch NIP was that it is a standard program: *“It's a pity that it's a standard program and there's little regard for the child's personal development,” *and that vaccinating is the general norm:* “One thing I don't like about it is that it's considered the norm to automatically or blindly follow the program, and that if you refuse vaccination, you have to justify your reasons.”*

### 3.3. Anthroposophical CWC

Because all of the parents in these focus groups visited an anthroposophical CWC, we were interested in why they had chosen an anthroposophical CWC and what their experiences were.

#### 3.3.1. Reasons to Visit an Anthroposophical CWC

Some parents (*n* = 6) visit an anthroposophical CWC because they have an anthroposophical background, lifestyle or beliefs: *“We chose an anthroposophical CWC because it was the obvious choice for us. As a child, I always went to an anthroposophical CWC, so it was part of my upbringing.”* Another parent said: *“We chose anthroposophical health care a long time ago because it has a different view of health, and it doesn't only look at physical aspects, but also spiritual aspects.”* Other parents (*n* = 5) said they visited an anthroposophical CWC because they had had a negative experience at a standard CWC: *“I first went to a regular CWC, but they were very strict and you almost felt guilty if you wanted to postpone or even considered postponing the vaccination*.*”*

#### 3.3.2. Experience at Anthroposophical CWC

All participants mentioned they had a positive experience when visiting an anthroposophical CWC. Most anthroposophical CWCs have longer consultations than standard CWCs and are therefore able to dedicate more time to informing the parents: *“The consultations are very pleasant, there's no pressure, the information you get is broad and clear and they make sure that you've really had the chance to think through the consequences (of not vaccinating). If you have any questions at a later time, you can always call.” *The parents also indicated that they appreciated it that health care workers at anthroposophical CWCs emphasize that parents have their own responsibility in making choices for their children:* “They (the health care workers) make it very clear that it's your own decision. They insist on nothing*.” 

### 3.4. Factors Influencing Decision-Making

The participants described various factors that influenced their choice whether to refuse or accept vaccinations for their children. These included lifestyle, perception of health, beliefs about childhood diseases, risk perception of the diseases, perceptions about vaccine effectiveness and vaccine components, and trust in institutions.

#### 3.4.1. Lifestyle

The parents indicated that the lifestyle they had might positively influence the health of their children. They tried to raise their children to be as healthy as possible so that their immune systems would be strong and able to cope with infectious diseases. One parent said: *“You can make sure your child is healthy and has a strong immune system. I think that's something I try to succeed in.”* Some parents (*n* = 5) indicated that a peaceful environment was important:* “We had a babysitter, our children did not went to day care, so they grew up in a quiet environment. This made me confident that I could postpone vaccination.” *Another parent said: *“They both went to childcare at a very late age, or we had a babysitter at home, so the care that our children get is good, there was a rest around them that supported them.”* Other parents (*n* = 6) mentioned that good nutrition such as breastfeeding was an important protector against infectious diseases: “*Because I breastfed for a long time I thought: ‘well my child will get a lot of protection from breast milk'.”*

#### 3.4.2. Perception of Health

The perception that the parents had about the health of their children was an important factor in their vaccination decisions: *“I look at how well she's developed. She's not weak, she's a very strong child and I have so much confidence in her. She's so healthy and I didn't want to interfere with that, so she hasn't been vaccinated.”* Most parents (*n* = 9) who refused vaccinations indicated that they had a lot of confidence in the health of their child: *“I have a lot of confidence in children's own healing power.”*

#### 3.4.3. Childhood Diseases

Some of the parents (*n* = 5) believe that certain diseases, so-called childhood diseases, are essential to the development of a child: *“According to anthroposophy, some childhood diseases contribute to your personal development, diphtheria, tetanus and polio are not part of that development and so we accepted this vaccine.”* Another parent said: *“I notice that the children are a bit listless when they have fever. They have fever for a few days, and then they make a sudden leap forward, or they start getting teeth. I've had childhood diseases myself. I had measles quite severely, but once it's over you've become stronger because you've overcome something.”* All parents refused the MMR vaccination, because they believe that the illnesses related to these vaccines are childhood diseases. Some of the parents (*n* = 3) expected their child to get the disease, but said they would reconsider vaccination if their children had not had the disease by a certain age: *“If they (children) have finished elementary school and they still haven't had MMR diseases, then we'll discuss the vaccination again. Because, well, at some point you're no longer a child.”*

#### 3.4.4. Risk Perception of Disease

The decision whether or not to vaccinate is also based on the perceived severity and susceptibility of the vaccine-preventable diseases. One parent said about vaccine-preventable diseases in general:* “If you take a look at the percentages, there's a very small number of children who have severe cases of it (the vaccine-preventable diseases).” *Some parents (*n* = 8) mentioned that they believe children are highly susceptible to tetanus and therefore vaccinated their child against it: *“Tetanus is the most important for me, because I think you can get that very easily.” *However, most parents (*n* = 13) mentioned that the perceived severity of the disease is important: *“We both had mumps, measles and rubella and we survived, so we don't vaccinate against MMR.” *Another parent said: *“I think diphtheria and polio are very severe diseases, even though the chance of getting them is very small, so I can imagine that we'll choose to vaccinate our daughter for that. We also vaccinated our sons against it.”*

#### 3.4.5. Vaccine Effectiveness

Some of the participants (*n* = 5) had doubts about the effectiveness of the vaccines: *“The graphs and reports I've read don't prove its (the vaccination's) effectiveness to me.”* Another parent said: *“I would like to ask the Public Health Institute (PHI) to show that vaccines are effective, this is never shown to me.” “I believe that they vaccinate with components that are not effective, that is a shame.”*

#### 3.4.6. Vaccine Components

Parents also had doubts about the components of the vaccines: *“It (the components) is all poison that you're injecting, so there's no positive component in a vaccination.” *Parents (*n* = 5) were also negative about combination vaccines, because they felt that their freedom of choice was limited (they could not choose separate vaccines) and because of a perceived overload for their child: *“I'm happy I didn't do it (vaccinate child with combination vaccine). How can you inject that into such a small child? And combining vaccines, just so you only have to give one injection … Then I think, do you really have such different views about it, like, well, it cannot hurt, or is it because of efficiency?” *Another parent said:* “I think that lots of parents would stop vaccinating if combination vaccines were the only vaccines offered. But if we're able to choose, I think more people will continue to vaccinate their children because they can select which vaccines they want.”*

#### 3.4.7. Trust in Institutions

There were different findings about trust in institutions. A few parents (*n* = 2) mentioned that if their child gets sick (because they refused vaccination) they would trust the health care in The Netherlands: *“I trust the health care in the Netherlands, but first and foremost I trust the health of my child.” *Other parents (*n* = 3) mentioned trust in the processes used to develop the vaccines: *“Well I have a lot of confidence in the technical process. I mean, I trust their account of what's in the cocktail, and that there aren't many other things in it and that it's been made very carefully and with the appropriate level of controls.” *However, parents (*n* = 7) did not always trust the information provided by the PHI:* “I have no confidence in their (the PHI) honesty or in them being open about how it works. I'd say the information is manipulated, not with bad intention, but because of their convictions.”*

### 3.5. Responsibility for Negative Consequences

This qualitative study showed that, whichever choice the parents make, they are willing to take responsibility for negative outcomes:* “You need a strong vision. What if your child gets polio. We thought ‘okay, we can deal with that. So we took that responsibility.”* Another parent said: *“Look, you cannot say as an anthroposophist, ‘well, we rely on the fact that polio has almost disappeared because most people are vaccinated'. The only thing you can say as an anthroposophist is: ‘if my child gets that disease, then that's his or her path, that's the path of development for that particular child'. So that's the risk you take.”*

### 3.6. Social Environment

The parents discussed the different experiences they had with their social environment. Sometimes their social environment, such as their family, influenced their thinking about vaccination: *“I once had a conversation with my mother about it and she said that I reacted badly to the vaccines, just like my brother and sister. Then I thought: ‘okay, if we reacted badly to the vaccines, maybe I shouldn't vaccinate my child just yet'.”* Most parents (*n* = 9) indicated that they did not tell other people in their social environment about their decision to refuse or postpone vaccination: *“My family, well they don't even think about it. They vaccinate their children and follow the general Dutch NIP. I don't talk with them about vaccination. I do my own thing.”* Sometimes parents chose not to discuss vaccination with others in their social environment because they had received negative reactions in the past: *“I got social support from my mother, not from the rest. (They said) I was irresponsible; they didn't want to talk about it.”* Another parent said: *“If I did the same as my social contacts, then I'd just do it (vaccinate) and take part in the general Dutch NIP. I always have to defend myself.”*

### 3.7. Information Need

A topic that was mentioned by all of the parents was their need for information. Different topics of information were mentioned. Almost all participants (*n* = 12) indicated they wanted more information about the risks of vaccinating: *“More information about the risks of vaccinating. Information that explains there are risks involved in getting vaccinated. There's not enough of that kind of information.” *The parents also wanted more scientific facts: *“I think that information should be objective and that means being complete, so including background on the disease, percentages of fatal cases, and so forth, so you know what the risks are. So information about the side effects as well, like the percentages of cases with side effects, just objective scientific measurable things.”* Other parents (*n* = 3) mentioned that they would like more information to be provided about the vaccine components in, for example, the vaccine leaflet: *“If I buy paracetamol at the drugstore, there's a leaflet included. Why not with vaccines?”*

Parents mentioned that despite the fact that they do not always trust the PHI, the institute should still provide parents with information about the Dutch NIP: “*Anybody can put information on the Internet, so I'd say the PHI is the best source for information about the Dutch NIP.”* It was mentioned and acknowledged by other parents in the focus group (*n* = 6) that if the PHIs were more transparent regarding the sources they used in their education material, they would be perceived as more reliable: “*It's important that the PHI lists the references for the information given in the education materials. By doing that, they would make the information more reliable.”*

## 4. Discussion

This study explored factors that influenced vaccination decision-making among parents with an anthroposophical worldview. Our findings show that parents in this study did not refuse all Dutch NIP vaccinations, but mostly refused the MMR vaccination. The participants made a deliberate decision whether or not to vaccinate. This is not in line with 81% of Dutch parents who are reported to make no comparative assessment of vaccinations [16]. The vaccination decisions of parents in this study are related to their lifestyle, perception of health, beliefs about childhood diseases, risk perception of the diseases, perceptions about vaccine components and vaccine effectiveness, and trust in institutions.

A previous study indicated that there is limited time for consultations at CWCs and therefore limited time to discuss childhood vaccination with parents [13]. This does not apply to parents who visit an anthroposophical CWC. All parents in this study indicated that they had positive experiences when visiting an anthroposophical CWC and mentioned that the consultations were longer and they were provided with more information. Some parents had had a negative experience at a standard CWC and indicated that standard CWCs are not flexible enough with regard to the Dutch NIP. These negative experiences may have resulted in their reluctance to accept the Dutch NIP and PHI guidance.

Parents in this study are not fully opposed to childhood vaccination. Mostly these parents refuse the MMR vaccination because they perceive measles, mumps, and rubella to be childhood diseases that are essential for the physical and mental development of their child. This is consistent with findings among anthroposophists by Duffell (2001) [10]. The rejection of the MMR vaccination is also reflected in lower vaccination coverage for the MMR vaccine [6–11] and outbreaks of measles among anthroposophists [6, 9–11]. Another factor that influences parents' choice whether or not to vaccinate is the low perceived risk of the vaccine-preventable diseases, which is also shown in other studies [17–19]. In addition, this study shows that the perceived severity of a disease seems to be more important than the perceived susceptibility to that disease. Parents indicated that they were aware of the possible negative outcomes of not vaccinating their child and were willing to take responsibility for those outcomes.

Most Dutch NIP vaccinations are combination vaccines. The parents were quite negative about the combination vaccines because of the perceived overload of the immune system (an anxiety also felt by parents who accepted all childhood vaccination [20, 21]) and because it limits their choices in vaccinating (parents can only vaccinate their children with combination vaccines and cannot choose separate vaccines). This point of view seems in line with wishes of anthroposophical CVPs to offer a flexible schedule and no combination vaccines [13], while CVPs at a regular CWC were positive about the combination vaccines because of their efficiency [13]. Some studies mentioned that another important factor for refusing vaccination is the side effects [2, 10]. The parents in our study were specifically concerned about the negative effects of the vaccine components. Besides that, some parents in this study had doubts about the efficacy of the vaccines. The findings above suggest that determinants that are associated with vaccination decision-making among anthroposophists are comparable to determinants held by parents in general. Only the view about healthy child development and childhood diseases distinguishes the parents in this study from parents in general. 

Another topic that is raised by different studies among parents who refuse vaccination is their information need [22–24]. Parents in this study indicated they need information about the risks of vaccination as well as the components and effectiveness of the vaccines, and that they would like to receive more detailed scientific information. Not only the parents would like more information, anthroposophical CVPs have also indicated that they need more information to better educate parents [13]. Parents mentioned the CVPs as the most important source of information about childhood vaccination [25, 26]. The focus should, therefore, not only be on providing information to parents, but also to CVPs, which would enable them to provide parents with more and better information. In addition, if parents' information needs are not fulfilled, they might start searching for information themselves, with the result that their vaccination decision-making may be influenced by widespread antivaccine messages [27–29]. Parents do not always trust the PHI, but the institute is still perceived as the most important, logical, and reliable information source about the Dutch NIP. To increase the reliability of the information provided by the PHI, references should be listed about the information sources used in their education material.

It should be mentioned that this study has some limitations. First, it might be that our study did not reach full saturation [30]. During the third focus group, no new themes emerged, compared to the former two focus groups, so we assumed that more focus groups were unnecessary but we did not test this by conducting a final focus group. The second limitation of our study is that we have no insight into the parents' demographic variables, like levels of education. A study by Hak et al. (2005) [31] showed that highly educated parents are more critical towards childhood vaccination. Third, there is potential for moderator bias in this study [32]. We tried to avoid this by the use of a standardized topic list, an assistant who was present at the focus groups, digital voice recorder, and verbatim transcriptions.

Further quantitative research on perceptions about the (Dutch) NIP is needed to be able to generalize results. The six themes that emerged from these results could be useful for developing quantitative research about parents, and anthroposophical parents in particular, who are critical of childhood vaccination. Quantitative research is also needed to get more insight in which determinants are most important in the decision making of parents with an anthroposophical worldview and whether these determinants are different from determinants influencing vaccine refusal in general.

## 5. Conclusion

This study showed that anthroposophical parents in this study are not opposed to vaccination in general. Their decision is not solely based on weighing the risks of vaccination against those of nonvaccination; it also depends on the parents' lifestyle and views about healthy child development. Parents in this study reported a need for information about childhood vaccination. However, not all parents want the same amount of information that these parents require. Layered information might therefore be an appropriate method to fulfill the information need of all parents. Sufficient references to sources containing more information about childhood vaccination should be provided, especially regarding the effectiveness of vaccines and vaccine components and the benefits and risks of vaccination, such as the possible side effects. This may satisfy parents' information needs and enable them to make a sufficiently informed choice whether or not to vaccinate their child. Further research is needed on how this information can best reach the parents who need it and if the information geared towards anthroposophical parents should be different from information geared towards parents that refuse childhood vaccination in general.
